# Epigenetic Profiles in Children with a Neural Tube Defect; A Case-Control Study in Two Populations

**DOI:** 10.1371/journal.pone.0078462

**Published:** 2013-11-05

**Authors:** Lisette Stolk, Marieke I. Bouwland-Both, Nina H. van Mill, Michael M. P. J. Verbiest, Paul H. C. Eilers, Huiping Zhu, Lucina Suarez, André G. Uitterlinden, Régine P. M. Steegers-Theunissen

**Affiliations:** 1 Department of Internal Medicine, Erasmus MC, Rotterdam, The Netherlands; 2 Department of Obstetrics and Gynecology, Erasmus MC, Rotterdam, The Netherlands; 3 Department of Child- and Adolescent Psychiatry, Erasmus MC, Rotterdam The Netherlands; 4 Department of Biostatistics, Erasmus MC, Rotterdam, The Netherlands; 5 Dell Pediatric Research Institute, Department of Nutritional Sciences, UT Austin, Austin, Texas, United States of America; 6 Division of Disease Control and Prevention Services, Department of State Health Services, Austin, Texas, United States of America; 7 Department of Epidemiology, Erasmus MC, Rotterdam, The Netherlands; 8 Department of Clinical Genetics, Erasmus MC, Rotterdam, The Netherlands; 9 Department of Epidemiology and Biostatistics, Radboud University Medical Center, Nijmegen, The Netherlands; The University of Tennessee Health Science Center, United States of America

## Abstract

Folate deficiency is implicated in the causation of neural tube defects (NTDs). The preventive effect of periconceptional folic acid supplement use is partially explained by the treatment of a deranged folate-dependent one carbon metabolism, which provides methyl groups for DNA-methylation as an epigenetic mechanism. Here, we hypothesize that variations in DNA-methylation of genes implicated in the development of NTDs and embryonic growth are part of the underlying mechanism. In 48 children with a neural tube defect and 62 controls from a Dutch case-control study and 34 children with a neural tube defect and 78 controls from a Texan case-control study, we measured the DNA-methylation levels of imprinted candidate genes (*IGF2*-DMR, *H19, KCNQ1OT1)* and non-imprinted genes (the *LEKR/CCNL* gene region associated with birth weight, and *MTHFR* and *VANGL1* associated with NTD). We used the MassARRAY EpiTYPER assay from Sequenom for the assessment of DNA-methylation. Linear mixed model analysis was used to estimate associations between DNA-methylation levels of the genes and a neural tube defect. In the Dutch study group, but not in the Texan study group we found a significant association between the risk of having an NTD and DNA methylation levels of *MTHFR* (absolute decrease in methylation of −0.33% in cases, P-value = 0.001), and *LEKR/CCNL* (absolute increase in methylation: 1.36% in cases, P-value = 0.048), and a borderline significant association for *VANGL* (absolute increase in methylation: 0.17% in cases, P-value = 0.063). Only the association between *MTHFR* and NTD-risk remained significant after multiple testing correction. The associations in the Dutch study were not replicated in the Texan study. We conclude that the associations between NTDs and the methylation of the *MTHFR* gene, and maybe *VANGL* and *LEKKR/CNNL*, are in line with previous studies showing polymorphisms in the same genes in association with NTDs and embryonic development, respectively.

## Introduction

Neural tube defects (NTDs) are congenital malformations accompanied with low birth weight, which result from a failure of the neural tube to close during the fourth week of embryogenesis. The most common phenotypes of NTDs are spina bifida and anencephaly [1]. Interactions between environmental exposures and subtle variations in genes are thought to be involved in the pathogenesis of NTD. A maternal folate shortage during the sensitive period of neural tube development contributes to the development of NTD, and the occurrence of the disease can be reduced by periconceptional maternal folic acid supplement use [2]. However, the mechanisms underlying this relationship are not completely understood.

Epigenetic alterations can cause changes in gene expression that are not directly related to the DNA sequence itself, of which DNA-methylation is one of the best understood epigenetic mechanisms [2]. DNA-methylation of genes is rather stable throughout life, except for example in the periconception period. It is an important mechanism in epigenetic programming of tissues, organ development and functioning. To establish DNA-methylation, DNA methyltransferases use methyl donors such as folate derived from the one carbon metabolism (1-C metabolism) essential during the periconceptional period [3]. Therefore, a reduced availability of methyl donors can lead to DNA hypomethylation with consequences for epigenetic programming. Several substrates like folate and cofactors such as vitamin B12 are implicated in 1C-metabolism. The 1C-metabolism has repeatedly been associated with DNA-methylation [4], [5]. Maternal vitamin B12 deficiency has been associated with an increased risk for NTD [6]–[10], linking maternal 1-C metabolism to closure of the neural tube in the developing child.

Higher DNA-methylation levels of *IGF2* DMR have been reported in children of mothers taking periconceptional folic acid in supplements [11]. High maternal homocysteine levels are associated with NTDs in the offspring [12] and with genome-wide hypo DNA-methylation in umbilical cord blood cells [13], [14]. In addition, vitamin B12 has also been suggested to influence DNA-methylation in umbilical cord blood cells [15]. Moreover, genetic variants in key genes that are involved in the 1C-metabolism are related to changes in DNA-methylation. Common variants of the *MTHFR* gene (c.677C>T and c.1298A>C) can result in a reduction of enzymatic activity [16], [17], which together with a low folate and or vitamin B12 status can result in elevated homocysteine levels and global DNA hypomethylation [18]–[20].

These *MTHFR* polymorphisms have been well studied in associations with NTD [16], [17]. Animal studies suggest that DNA-methylation is implicated in neural tube closure [21], [22], indicating that changes in DNA-methylation could influence the risk for NTD. Recently, it was shown by Chang et al. that maternal serum folate was lower in NTD cases compared to controls, and that the maternal serum folate levels were correlated with methylated cytosines in the brain of NTD fetuses [23]. A previous study reported hypomethylation of the DNA-repair gene *MGMT* in brain tissue of NTD cases [24]. Global hypomethylation was also found to be association with NTD in brain tissue [25] as was LINE1 hypomethylation [26]. From this background, we hypothesize that derangements in DNA-methylation of (non)imprinted genes implicated in embryonic and neural tube development contribute to NTD risk. In two cohorts of NTD cases and controls, we studied associations between DNA-methylation levels in fetuses and very young children using a candidate gene approach of imprinted candidate genes (*IGF2*, *H19, and KCNQ1OT1)* and non-imprinted *LEKR/CCNL* gene region involved in embryonic development and *MTHFR*, and *VANGL1* that are associated with NTDs.

## Methods

### Study Design and Data Collection

#### Dutch case-control study

Between August 1999 and December 2001, a case-control triad study was conducted in which mothers of a child with a non-syndromatic meningo(myelo)cele, i.e., spina bifida, were enrolled in collaboration with the Dutch Spina Bifida Teams of the University Medical Centers of Nijmegen, Utrecht, Groningen, Rotterdam, Leiden, Amsterdam, and the regional hospitals in Tilburg and Zwolle in The Netherlands. Dutch Caucasian women and their children between 1 and 3 years of age were eligible to participate. The spina bifida was diagnosed by a neuropediatrician at birth. Control subjects were recruited from acquaintances and nurseries in Nijmegen and surroundings in the Netherlands as described in detail previously [6], [27]. Written informed consent was obtained from the parents of the children. The informed consent and study protocol was approved by the Institutional Review Board of the University Medical Center Nijmegen, the Netherlands.

The original study population comprised 63 mothers and 70 children with spina bifida and 102 control mothers and 85 children. In the present study we selected 48 case and 62 control children with available high quality DNA derived from pheripheral white blood cells. For standardized research data collection parents and their children visited the outpatient clinic of the University Medical Center Nijmegen, the Netherlands. Mothers filled out questionnaires concerning demographic data and lifestyle factors in the periconceptional period (3 months before until 3 months after conception) of the pregnancy and at the moment of blood sampling [3]. Maternal body weight and length were measured to calculate the body mass index (BMI, kg/m^2^). During the visit 2 ml of maternal venous blood and 1 ml of venous blood of the child were drawn to measure concentrations of red blood cell (RBC) and serum folate, serum vitamin B12 and plasma total homocysteine (tHcy) and handled as described previously [6]. Maternal blood samples were drawn after an overnight fast. Children’s white blood cell genomic DNA was extracted from whole blood according to standard procedures [28].

#### Texan case-control study

Study subjects were recruited from a Mexican American population from the 14 counties along the Texas-Mexico border region that terminated their pregnancies or delivered between 1995 and 2000. Cases were terminations (spontaneous or selective abortions), livebirths, or stillbirths with any diagnosis of NTD, defined as spina bifida or anencephaly. They were identified through hospitals, birth centers, ultrasound centers, abortion centers, prenatal clinics, genetics clinics, and birth attendants (midwives and non-hospital physicians). Controls were non-malformed live births that occurred in the same counties during that time period. Subjects were approached about study enrollment either prenatally or in the hospital at the time of delivery or pregnancy termination. Interviews were scheduled approximately 1 month postpartum. Before the interview, written informed consent was obtained from both parents of the subject in the parent’s preferred language. The protocol, consent forms, and questionnaire were approved by the Texas Department of Health institutional review board for the protection of human subjects [29]. The study protocol was reviewed and approved by the Texas A&M University System Health Science Center Internal Review Board (IRB) and the Department of State Health Services (formerly Texas Department of Health) IRB. Participant identification and data collection have been described in detail elsewhere [9], [30], [31].

In the present study 34 cases and 78 control children were included. Women were interviewed at home using a standard instrument modeled after the Centers for Disease Control and Prevention’s 1993 mother questionnaire for birth defects risk factor surveillance. With this instrument, information was obtained on maternal age at conception, years of schooling, maternal folic acid supplement use and smoking habits during the periconceptional period (from 3 months before to 3 months after conception). BMI was calculated from self-reported pre-pregnancy height and weight. During the interview, maternal blood samples were drawn to measure concentrations of red blood cell (RBC) and serum folate, and serum vitamin B12 and handled as described previously [9].

Genomic DNA was extracted from 5 2.5 mm dried blood spots (Guthriepaper) using the Quickgene SP DNA tissue kit (Fujifilm) according to the manufacturers protocol. Isolated DNA was stored at −20°C.

### Quantitative Assessment of DNA-methylation

The amplicons for *IGF2-DMR, H19* were described previously [32]. Amplicons for *KCNQOT1*, *MTHFR, VANGL1* and *LEKR*/*CCNL* were designed using the EpiDesigner tool (Sequenom, Inc, San Diego, USA). The three imprinted genes are *IGF2*-DMR, *H19* and *KCNQOT1*, which are involved in embryonic growth and development. The three non-imprinted genes are *MTHFR*, *VANGL1* and *LEKR/CCNL*. *MTHFR* is involved in 1-C metabolism and the 677C>T and 1298A>C polymorphisms in this gene as well as mutations in *VANGL1* are associated with the risk of NTD [16], [17], [33]–[35]. We also selected the candidate gene-region *LEKR*/*CCNL*. A polymorphism in the same LD-block as the CpG-island in this region is associated with birthweight, a marker often used for prenatal growth [36], Amplicons for IGF2-DMR and H19 were taken from the study by Heijmans et al [37]. Table S1 shows the location, length and primers of the amplicons. First, the amplicons were tested on a standard curve constructed from DNA with low and high methylation (EpigenDx, Worcester, MA, USA) in steps of 10% methylation difference. Only amplicons with a good distribution of the methylation percentages were used for the measurements of the samples. In Figure S1 the locations of the CpG-islands studied with respect to the genes are depicted.

Isolated genomic DNA (500 ng) was treated with sodium bisulphite for 16 hours using the EZ-96 DNA-methylation kit (Shallow) (Zymo Research, Irvine, CA, USA). This was followed by PCR amplification, fragmentation after reverse transcription and analysis on a mass spectrometer (Sequenom, Inc, San Diego, USA). This generated mass signal patterns that were translated into quantitative DNA-methylation levels of different CpG sites of earlier mentioned genes in all tissues by MassARRAY EpiTYPER Analyzer software (v1.0, build1.0.6.88 Sequenom, Inc, San Diego, USA) [38]. Fragments containing one or more CpG sites were called CpG units. Measurements were done in triplicate on DNA from the same bisulfite-treatment batch on different PCR-plates. On every bisulphite plate, standard DNA with low methylation, 25%, 50%, 75% and high methylation was included. This standard DNA was used to check the technical steps of the experiment.

### Data Cleaning

During quality control CpG units with a too low or too high mass or CpG unitswith overlapping RNA fragments were excluded from further analysis. Furthermore, two out of three of the replicate measurements per CpG unit had to be successful, and the SD of the duplicates or triplicates had to be ≤0.10. Outliers per CpG (>3SD) were excluded from further analysis, CpG units with interference of SNP’s were also excluded. Bisulfite conversion was assessed using the MassArray R package [39], which uses fragments containing a TpG and a cytosine to assess the conversion. This showed >99% bisulfite conversion.

### Statistical Analysis

To test for differences in maternal or child characteristics between cases and controls, X^2^-, t- and Mann-Whitney U tests were used. The mean methylation fractions presented in the tables are based on raw data. ANOVA was used to detect possible batch-effects of bisulphite-plate and PCR plate. For analysis of differences in total methylation per amplicon between cases and controls, linear mixed models were applied. Linear mixed models were chosen as these models allow for the analyses of multiple CpG dinucleotides in one test, account for correlation between CpG dinucleotides, incorporate relevant adjustments within the models and have the ability to accommodate missing data. The REML likelihood method was used for the model fitting. DNA-methylation was treated as a continuous variable. All models account for the correlation between CpG dinucleotides, and bisulphate plate number. The final analysis takes into account potential confounding variables, including infant age and maternal education (Dutch study) and maternal education (Texas study) which were all entered as fixed effects. Person identifier was entered as random effect.

Next, a possible mediating effect of DNA-methylation in the association between maternal biomarker concentrations and NTD risk was tested. As extended information was available of the Dutch population, these analyses were only performed within this study. First, we created a standard deviation score (SDS) for maternal biomarkers levels as the absolute differences were minor. We assessed the association between serum folate, vitamin B12 and total homocysteine (tHcy) and the occurrence of NTD using multivariable logistic regression models. The mean *MTHFR* methylation was added to the latter model to assess possible mediation.

All tests were performed using the data measured in triplets and all reported P-values are 2-sided. Effect sizes for NTD were expressed in relation to the SD in the controls. To account for multiple testing the Benjamini-Hochberg FDR correction was used. All analyses were performed using SPSS software, version 17.0 (IBM SPSS Inc. Chicago, IL, USA).

## Results

The two cohorts in this study are a Dutch case-control study with surviving spina bifida cases, and a Texan study with both alive and deceased spina bifida and anencephaly cases. Next to the difference in cases of both studies the main difference is the age of the children at investigation. The Dutch cases and controls were investigated at the standardised age of approximately 15 months, while the Texan study groups were investigated after pregnancy termination or birth at the time of the Guthrie test.

The characteristics of the cases and controls are shown in Table 1. Dutch case mothers showed a higher BMI (+2.4 kg/m^2^, P-value = 0.011), a lower maternal education (20% less high education, P-value = 0.021) and lower serum vitamin B12 levels (−86 pmol/L, P-value = 0.007) compared to control mothers. Children with NTD had a slightly higher age at blood collection (+0.5 y, P-value<0.001), and lower birthweight (−230 g, P-value<0.0001) and shorter gestational age at birth (−1.1 week, P-value = 0.001) compared to controls. Whereas case mothers of the Texan study only had a significantly higher BMI compared to control mothers (+1.8 kg/m^2^, P-value = 0.035). Characteristics of the Texan children were not available for analysis.

**Table 1:** Baseline characteristics for the two study groups.

	Dutch study group			Texan study group		
Characteristics	NTD children	Control children		NTD children	Control children	
	(n = 48)	(n = 62)	P-value	(n = 40)	(n = 79)	P-value
Maternal						
Age at delivery (y)*	30.5 (3.9)	31.5 (3.3)	NS	24.8 (6.0)	24.8 (6.1)	NS
BMI (kg/m2)^†^	25.4 (7.5)	23.0 (4.2)	0.011	26.5 (8.1)	24.7 (7.4)	0.035
Education: high, n (%)	6 (12.8)	20 (33.3)	0.021	–	–	–
Education (years of school)^†^	–	–	–	11 (5)	12 (4)	NS
Periconceptional folic acid supplement use:yes, n (%)	31 (66.0)	38 (63.3)	NS	3 (7.5)	2 (2.6)	NS
Periconceptional smoking: yes, n (%)	12 (25.5)	15 (25.0)	NS	9 (22.5)	10 (12.8)	NS
tHcy, plasma (µmol/L)^†^	10.6 (3.3)	11.1 (4.7)	NS	–	–	–
Folate, serum (nmol/L)^†^	16.3 (15.9)	13.4 (13.2)	NS	25.4 (23.6)	22.9 (31.3)	NS
Folate, RBC (nmol/L)^†^	780 (665)	595 (552)	NS	738 (616)	824 (555)	NS
Vitamin B12, serum (pmol/L)^†^	227 (152)	313 (184)	0.007	323 (140)	387 (162)	NS
Child						
Age at the study moment (y)^†^	1.7 (1.5)	1.2 (0.3)	<0.001	–	–	–
Male sex, n (%)	16 (34.0)	24 (40.0)	NS	–	–	–
Birth weight (g)*	3113 (552)	3343 (313)	<0.001	–	–	–
Gestational age at birth (weeks)^†^	38.9 (2.2)	40.0 (1.3)	0.001	–	–	–
tHcy, plasma (µmol/L)^†^	6.3 (3.1)	7.1 (1.3)	NS	–	–	–
Folate, RBC (nmol/L)^†^	1086 (752)	1228 (813)	NS	–	–	–
Folate, serum (nmol/L)^†^	31.5 (28.3)	31.0 (47.6)	NS	–	–	–
Vitamin B12, serum (pmol/L)^†^	503 (335)	471 (282)	NS	–	–	–

Data is presented of the Dutch study group (n = 110) and the Texan study group (n = 119). Values are presented as * mean (SD) or ^†^ median (IQR). Chi-square, t- and Mann-Whitney U tests were used to test differences between NTD and control children.

**Figure 1:**
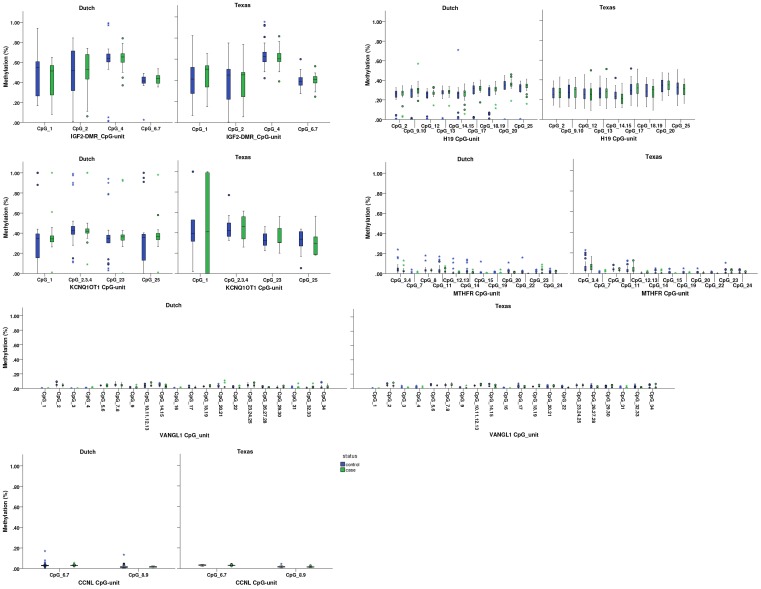
Methylation levels per locus stratified by study and case-control status. Box and whisker plots of methylation values (y-axis) of all individuals are shown for each CpG unit (x-axis) for NTD case and control children separately.

The mean absolute DNA-methylation in the child according to case-control status is shown in Table 2 and Figure 1. The mean methylation presented in Figure 1 is stratified by CpG unit, study and case-control status. Effects and P-values in Table 2 are corrected for CpG, bisulfite treatment plate, maternal education (Dutch and Texan study samples) and age of the child (in the Dutch study only). In the Dutch study, the absolute methylation of *MTHFR* was 0.33% lower in case children compared to controls (P-value = 0.001). Methylation of *VANGL1* was 0.17% (P-value = 0.063) higher and of LEKR/CCNL 1.36% higher (P-value = 0.048) in spina bifida cases as compared to controls. After Benjamini-Hochberg correction for multiple testing only the association with *MTHFR* remained significant.

**Table 2:** Methylation in NTD and control children in the Dutch and Texan study groups.

	Ncases	Ncontrols	Control methylation % (SD)	Absolute difference	P-value
Dutch children					
* IGF2*-DMR	43	35	51.2 (22.1)	+0.96	0.494
* H19*	43	27	26.1 (11.9)	+0.62	0.333
* KCNQ1OT1*	41	54	38.7 (20.9)	−0.21	0.886
* LEKR/CCNL*	46	61	2.6 (2.4)	+1.36	0.048
* MTHFR*	47	61	1.2 (2.5)	−0.33	0.001#
* VANGL1*	47	59	2.0 (2.9)	+0.17	0.063
Texan children					
* IGF2*-DMR	32	67	46.1 (17.9)	+0.69	0.498
* H19*	24	50	28.2 (9.4)	−0.27	0.616
* KCNQ1OT1*	11	22	38.4 (18.0)	−0.49	0.890
* LEKR/CCNL*	22	48	2.5 (1.1)	−0.15	0.084
* MTHFR*	22	48	1.2 (2.3)	−0.07	0.470
* VANGL1*	33	78	1.9 (2.3)	+0.07	0.149

Linear Mixed Model analysis.

Corrected for CpGs, bisulfite treatment plate, age of the child (Dutch children only), and maternal education.

# P<0.05 after multiple testing correction by Benjamini-Hochberg FDR.

In the Texan study of all six studied genes only *LEKR/CCNL* showed a borderline significant association with NTD risk (effect: −0.15, P-value = 0.08), however, in the opposite direction from the results in the Dutch study group. So, we could not replicate the associations found in the Dutch study.

The associations between maternal biomarker concentrations and the risk of spina bifida were tested. These analyses were restricted to the Dutch population as these data were not available for the Texas population. We found that for each standard deviation increase of maternal vitamin B12 levels, a reduced risk (adjusted OR 0.992, 95%CI: 0.99–1.00, P-value = 0.050) for NTD was observed. Subsequently, the model was additionally adjusted for *MTHFR* methylation, to test for a mediation effect. The odds ratio was attenuated and no longer significant (adjusted OR 0.995, 95%CI: 0.99–1.00, P-value 0.27) (Table 3). However, *MTHFR* methylation itself was not significant in the model (P-value = 0.77, data not shown).

**Table 3:** Maternal biomarker concentrations and NTD risk in offspring in the Dutch study.

	NTD		
	aOR	95% CI	P-value
Model 1: adjusted for age of the child at blood sampling, maternal educational level,and folic acid supplement use at the moment of blood sampling			
Maternal folate SDS, serum (nmol/L)	0.785	0.118–5.221	0.802
Maternal tHcy SDS, plasma (µmol/L)	1.133	0.495–2.591	0.767
Maternal vitamin B12 SDS, serum (pmol/L)	0.380	0.145–0.999	0.050
Model 2: additionally adjusted for mean *MTHFR* methylation			
Maternal folate SDS, serum (nmol/L)	1.115	0.130–9.536	0.921
Maternal tHcy SDS, plasma (µmol/L)	1.158	0.499–2.686	0.733
Maternal vitamin B12 SDS, serum (pmol/L)	0.535	0.177–1.619	0.268

aOR, adjusted odds ratio.

Result from multivariable logistic regression analyses.

## Discussion

In this combined study of a Dutch and a Texan case-control study, we found in the Dutch study group an association of DNA-methylation levels of the *MTHFR* CpG-island with NTD in children. In addition, our results suggest mediation of *MTHFR* methylation in the association between maternal vitamin B12 and spina bifida in offspring. This association was not observed in the Texan study group. We did not find significant associations for the imprinted *IGF*2 DMR, *H19*, and *KCNQ1OT1* genes or for the non-imprinted genes *VANGL1* and *LEKR/CCNL* after correction for multiple testing.

The role of the folate dependent 1-C metabolism in the etiology of NTD has been consistently shown. Maternal hyperhomocysteinemia as a sensitive marker of a deranged 1-C metabolism has been associated with an increased risk of NTD in the offspring [12], [40], [41]. One of the proposed underlying mechanisms of this association is the induction of alterations in DNA-methylation of genes implicated in embryonic and neural tube development. DNA methyltransferases (DNMTs) use methyl donors from the 1-C metabolism to establish and maintain DNA-methylation [42]. Disruption of DNMT3b in animal models, which is responsible for *de novo* methylation, resulted in NTD [22], which supports DNA-methylation as possible mechanism in the causation of NTD. A key gene within the 1C-metabolism is *MTHFR*. Common variants of the *MTHFR* gene have been shown to be also associated with NTD risk [9], [20]. The combined heterozygozity of these two polymorphisms resulted in reduced activity of the enzyme and was associated with NTD [17]. In the present study, we found a small difference but significantly lower *MTHFR* DNA-methylation levels in Dutch children with spina bifida. Lower DNA-methylation levels could indicate higher activity of the gene, which would suggest an opposite effect from the study by Van den Put et al., where lower activity of the gene was associated with higher NTD risk [17]. Because we conducted a cross-sectional study we cannot distinguish whether the observed association is a cause or outcome of NTD. The samples in the Dutch study were drawn around 15 months after birth. Therefore, we cannot exclude an environmental or disease-driven effect on the DNA-methylation levels of *MTHFR* in these children.

The Dutch NTD have previously been associated with higher zinc, glutamine, lower glucose, and creatinine concentrations in amniotic fluid of the children [43], [44], and with higher glucose, lower zinc, myo-inositol and vitamin B12 concentrations in the mothers [6], [27], [45]. The lower maternal B12 concentrations in cases could lead to a derangement of 1C-metabolism, which could have effected DNA-methylation levels of *MTHFR* in the children. This could indicate that the lower *MTHFR* DNA-methylation level is implicated in the causative pathway of the development of NTDs. Nevertheless, *MTHFR* methylation was not significant in the mediation model. This could be due to the relatively small sample size of our study, so additional studies are warranted to confirm these results.

While we do not observe any association of *H19* with NTDs in blood of these subjects, a recent study in brain tissue of Chinese NTD cases and controls showed a significant decrease in *H19*-DMR1 DNA-methylation in cases [46]. One of the reasons that we find different results for H19 DNA-methylation in association with NTDs is that we studied a different region 5′of the H19 gene. Liu et al. studied a region located around 2 kb upstream of the gene, while the region we studied was located 300–700 bp 5′of the gene. Methylation at different regions at a locus could show uncorrelated results.

Some strengths and limitations of this study have to be addressed. Our study is the first to investigate DNA-methylation levels in peripheral white blood cells in relation to the occurrence of NTD in children. Previous studies on DNA-methylation levels in NTD studied DNA-methylation in brain tissue, but neither candidate gene nor global DNA-methylation in white blood cells [24]–[26]. In the present study we were not able to study brain tissue of NTD cases and controls, and therefore we are not sure if DNA methylation status of white blood cells is an appropriate surrogate for that of brain tissues during development. In particular, there is a possibility that the differences in DNA-methylation could be attributed to the cellular heterogeneity in leukocytes. Nonetheless, the ENCODE project showed recently that CpGs in promoters and upstream regulatory regions display less variation between cell types and tissues compared to CpGs in gene bodies and intergenic regions [47]. In the present study we have mainly focused on CpGs in CpG-islands in the promoter and upstream regions of the genes. Therefore, we expect that the levels we measure in whole blood are similar to those in brain tissue, or at least that the levels show moderate to good correlation. It remains unclear to what extend our results in white blood cells are comparable to brain tissue. However, it is impossible to perform these studies on human brain tissue in similar cases and controls. Although it is difficult to generalize our results, they might give us a clue to what genes are epigenetically involved in the development of NTDs.

Another strength of our study is that with the EpiTYPER method we have covered a great part of the CpG-island in the promoter or regulatory region of each gene, so we have conducted a very thorough search for the candidate genes we selected. Furthermore, we have compared data for two independent studies for the association of DNA-methylation with NTD, although we could not replicate our findings.

One of the limitations is the relative small control group for both the Dutch and the Texas study. For the most optimal power one would need 3 controls for each case and we only had one to two controls per case. However, based on previous studies we assume the effect of small DNA-methylation differences to be relatively large [5]. So we expect to identify true significant associations with our sample size and case:control ratio. The blood of the Texan study was obtained at the time of the Guthrie test, shortly after birth or after pregnancy termination while the blood of the Dutch study was obtained 15 months after birth. Therefore, due to this time difference we could not combine the Dutch and the Texas study samples, because we assume the 15 months difference in age to have an effect on the DNA-methylation levels of the children. Also, the Dutch cases are all spina bifida cases surviving one year of age, while the Texas samples include both spina bifida and anencephaly cases of which not all were survivors. For genetic variation it is well known that a different ethnic background could influence the linkage disequilibrium and therefore the association with outcomes. This is not known for epigenetic variations, which we could not study in our study populations.

A technical limitation of this study is the sensitivity of the method we have used to detect DNA-methylation levels. It is known for the MassARRAY technique that this is around 5%, while part of our results are lower than this. Therefore, additional studies using a different technique are needed to validate the observed association of *MTHFR* DNA-methylation with the risk for NTDs.

Lastly, we performed a candidate gene approach to investigate the effect of DNA-methylation in association with NTD, however, the evidence based selected genes may not be the main genes involved in NTD. So, we might have missed DNA-methylation differences between cases and control in other more important genes. To circumvent this, a hypothesis-free epigenome-wide association approach should be followed. However, this requires ample amounts of DNA, and because we studied DNA of children we did not obtain enough DNA from the small amounts of blood to perform these experiments in the present study.

This study was the first to investigate DNA-methylation levels of several imprinted and non-imprinted candidate genes in WBC in association with NTD. We observed a small but significant association of *MTHFR* methylation levels with NTD, which could support a potential functional link between 1C-metabolism and NTDs. This link should be investigated in other studies and more in depth in animal studies for example in order to establish causality of lower *MTHFR* DNA-methylation in NTD.

## Supporting Information

Figure S1 Location of the Sequenom amplicons tested with respect to *IGF2* (A), *H19* (B), *KCNQ1OT1* (C), *MTHFR* (D), *VANGL1* (E), *LEKR1-CCNL1* (F). Amplicons are depicted in black bars with arrows, genes are depicted in blue, CpG-islands are depicted in green. Figures are created in the UCSC Genome Browser (build hg19).
(DOCX)

Table S1 Details of measured amplicons and PCR primers.
(DOCX)
