# Correction: Epigenetic Profiles in Children with a Neural Tube Defect; A Case-Control Study in Two Populations

**DOI:** 10.1371/annotation/930bd671-19cc-4846-8352-bf9cdf11d6c9

**Published:** 2014-01-03

**Authors:** Lisette Stolk, Marieke I. Bouwland-Both, N. H. van Mil, Michael M. P. J. Verbiest, Paul H. C. Eilers, Huiping Zhu, Lucina Suarez, André G. Uitterlinden, Régine P. M. Steegers-Theunissen

The name of the third author was spelled incorrectly. The correct name is: N.H. van Mil. The correct Citation is: Stolk L, Bouwland-Both MI, van Mil NH, Verbiest MMPJ, Eilers PHC, et al. (2013) Epigenetic Profiles in Children with a Neural Tube Defect; A Case-Control Study in Two Populations. PLoS ONE 8(11): e78462. doi:10.1371/journal.pone.0078462. 

