# Supplementary material for: Epigenetic Profiles in Children with a Neural Tube Defect; A Case-Control Study in Two Populations
Source: PLoS One. 2013 Nov 5;8(11):e78462. doi: 10.1371/journal.pone.0078462 (PMC3818348; doi:10.1371/journal.pone.0078462)

**Supplementary Figure S1**. Location of the Sequenom amplicons tested with respect to *IGF2* (A), *H19* (B), *KCNQ1OT1* (C), *MTHFR* (D), *VANGL1* (E), *LEKR1-CCNL1* (F). Amplicons are depicted in black bars with arrows, genes are depicted in blue, CpG-islands are depicted in green. Figures are created in the UCSC Genome Browser (build hg19).

A


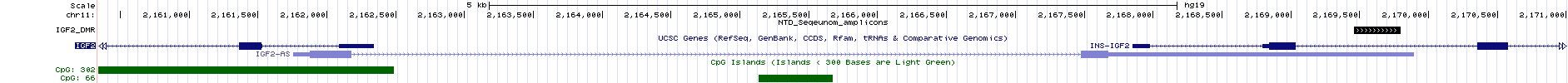


B


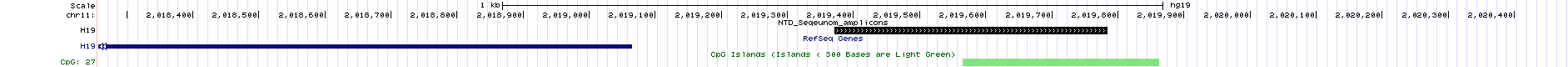


C


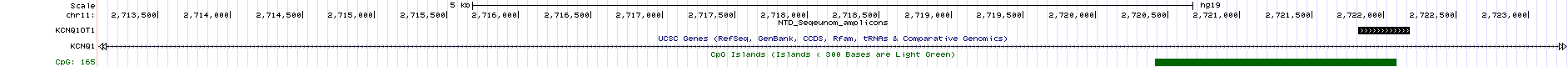


D


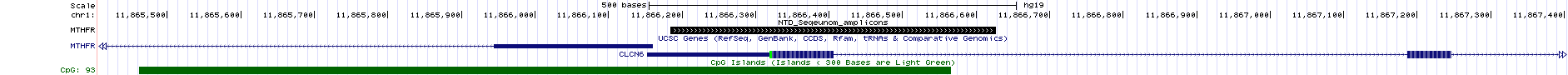


E


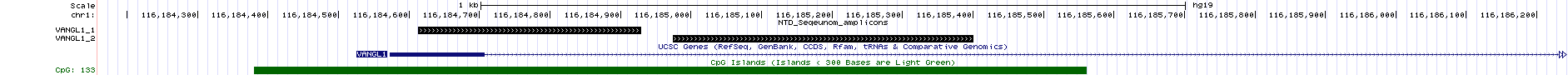


F


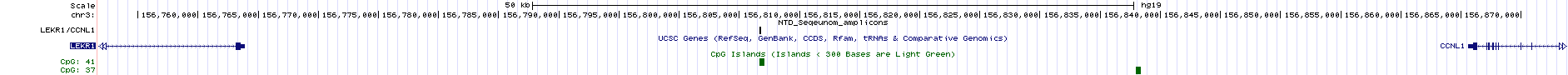

Supplement: Figure S1 — Location of the Sequenom amplicons tested with respect to IGF2 (A), H19 (B), KCNQ1OT1 (C), MTHFR (D), VANGL1 (E), LEKR1-CCNL1 (F). Amplicons are depicted in black bars with arrows, genes are depicted in blue, CpG-islands are depicted in green. Figures are created in the UCSC Genome Browser (build hg19). (DOCX) [file pone.0078462.s001.docx]
