# Supplementary material for: Epigenetic Profiles in Children with a Neural Tube Defect; A Case-Control Study in Two Populations
Source: PLoS One. 2013 Nov 5;8(11):e78462. doi: 10.1371/journal.pone.0078462 (PMC3818348; doi:10.1371/journal.pone.0078462)
Supplement: Table S1 — Details of measured amplicons and PCR primers. (DOCX) [file pone.0078462.s002.docx]

**Supplement table S1: Details of measured amplicons and PCR primers**

| **Gene** | **Genomic location^1^** | **Number of CpG units assessed** | **Primer sequence^2^** | **Source** |
| --- | --- | --- | --- | --- |
| *IGF2-DMR* | Chr 11: 2169458-2169796 | 3 CpG units  (4 CpG sites) | F: TGGATAGGAGATTGAGGAGAAA | Heijmans 2007 [[33](#_ENREF_33)] |
|  |  |  | R: AAACCCCAACAAAAACCACT |  |
| *H19* | Chr 11: 2019371-2019784 | 10 CpG units  (13 CpG sites) | F: GGGTTTGGGAGAGTTTGTGAGGT | Heijmans, 2007 [[33](#_ENREF_33)] |
|  |  |  | R: ATACCTACTACTCCCTACCTACCAAC |  |
| *KCNQ1OT1* | Chr11: 2721823-2722182 | 4 CpG units  (6 CpG sites) | F: GTTAGGGAAGTTTTAGGGTGTGAAT  R: TTCTAAAACCCCCACTACTATACCT | Designed using the Epidesigner tool^3^ |
| *MTHFR* | Chr 1: 11866184-11866627 | 12 CpG units  (14 CpG sites) | F: GTTTGTAGTTATTTTTGGTTTTAGTTTT | Designed using the Epidesigner tool^3^ |
|  |  |  | R: TAACCTAAATTCTCCCTCAAATTCC |  |
| *VANGL1*, primerset #1 | Chr 1: 116184613-116184929 | 8 CpG units  (15 CpG sites) | F: GAGAAGAGTGGAGTTAGAGGAAGTATTATT | Designed using the Epidesigner tool^3^ |
|  |  |  | R: ACTCTACCTCTCCAAAAACCCAAC |  |
| *VANGL1*, primerset #2 | Chr 1: 116184974-116185401 | 12 CpG units  (20 CpG sites) | F: AGTAGGGATATTTTGGGTAGAGATT | Designed using the Epidesigner tool^3^ |
|  |  |  | R: CACCCCACTCCTTAAAATCC |  |
| Region of *LEKR1* and *CCNL1* | Chr 3: 156806655-156806812 | 2 CpG units  (4CpG sites) | F: GTAAGGTTTTTGGGAAAGTTGTTTT | Designed using the Epidesigner tool^3^ |
|  |  |  | R: CTCTAAAACCCTCCCCTACCTC |  |

^1^ Genome built: GRch 37.67

^2^ Forward and reverse primer that will amplify the bisulphite converted genomic DNA. Primers were delivered with standard Sequenom MassCleave tags. Forward primer: 10mer spacer tag is added at the 5’ primer end with the following sequence: 5’-AGGAAGAGAG + primer. Reverse primer: T7 promoter is added to the 5’ primer end with the following sequence: 5’-CAGTAATACGACTCACTATAGGGAGAAGGCT + primer

^3^ Sequenom Inc, San Diego, USA
